# Evaluating stress antagonists for enhanced coral recovery after natural heat exposure

**DOI:** 10.1038/s41598-025-22120-0

**Published:** 2025-10-09

**Authors:** Selma D. Mezger, Yusuf C. El-Khaled, Susana Carvalho, Raquel S. Peixoto, Christian Wild

**Affiliations:** 1 Marine Ecology Group, Faculty of Biology and Chemistry, University of Bremen, Leobener Str. 6, 28359 Bremen, Germany; 2 Biological and Environmental Sciences and Engineering Division (BESE), King Abdullah University of Science and Technology (KAUST), 23955 Thuwal, Saudi Arabia

**Keywords:** Climate change, Coral physiology, Reef management, Stress mitigation, Short-term interventions, Nutrient dynamics, Ecophysiology, Restoration ecology, Tropical ecology

## Abstract

Hard corals are highly susceptible to heat stress, often leading to bleaching and mortality, making it critical to identify solutions for stress mitigation and recovery. We evaluated the effects of four potential stress antagonists (hydrogen, phosphate, ammonium, and probiotics) on the widespread hard corals *Acropora* spp. and *Pocillopora favosa* in short-term experiments following the 2023 heat stress event in the Central Red Sea. Corals were exposed to each treatment and a control for 48 h, thereby measuring survival, coloration, and photosynthetic efficiency. Hydrogen increased survival by 25% for *Acropora* spp. and 40% for *P. favosa*. Phosphate and ammonium both increased survival by 40% for both species, while enhancing photosynthetic efficiency and coloration of *P. favosa*. Probiotics increased survival by 20% for *Acropora* spp. and 40% for *P. favosa*, while improving photosynthetic efficiency for both species. Phosphate and ammonium addition resulted in 100% survival for *Acropora* spp.. These findings highlight the potential of all these stress antagonists in aiding coral recovery after heat stress. The use of these stress antagonists may be considered in reef and waste water management depending on the needs, logistics, and expertise available at each site.

**Supplementary Information:**

The online version contains supplementary material available at 10.1038/s41598-025-22120-0.

## Introduction

Coral reef ecosystems are not only essential for marine biodiversity but also serve as a vital source of food and income for millions worldwide^1–3^. Yet, they are exposed to numerous anthropogenic stressors, with climate change being one of the most influential ones^4,5^. Climate change-induced marine heatwaves (MHWs), occurring with increasing frequency and severity, have emerged as a major threat to coral reefs, causing widespread bleaching and mortality events^5–7^. The alarming rate of coral loss, with half of the world’s reef-building corals lost since 1950, highlights the urgent need for conservation efforts^8^.

The Red Sea, experiencing rapid warming, provides a stark example of the global trend, with temperatures rising faster than the global average^9,10^. As MHWs intensify, the frequency and magnitude of coral bleaching events are expected to increase, jeopardizing the resilience of coral reef ecosystems worldwide^11,12^. Understanding the resilience of corals, encompassing both their resistance to bleaching and recovery from it, is fundamental in assessing the overall ability of coral species to withstand and bounce back from environmental stressors^13,14^. Coral bleaching, for example, triggered by heat stress, breaks down the vital symbiotic relationship between corals and their zooxanthellae algae, leading to the loss of pigmentation and, eventually, coral mortality^15,16^. While some corals can recover from bleaching events, the extent and mechanisms of recovery vary among species and environmental conditions^17^.

Recovery is a complex process influenced by factors such as energy reserves, heterotrophic feeding capacity, and the resilience of symbiotic associations^18,19^. However, the capacity for recovery may be compromised by the increasing frequency of bleaching events, outpacing the natural recovery processes of coral reefs^18,20^. Previous research efforts have predominantly examined coral recovery over varying timescales, mainly from months to years, with a focus on parameters such as hard coral cover, symbiosis re-establishment, and energy reserve replenishment^14,18,21–23^. Also, these recovery studies have largely been conducted in situ under natural conditions without considering potential stress antagonists that could facilitate recovery. Moreover, the results of coral recovery studies have been diverse, particularly when considering annual bleaching events versus single occurrences. For instance, while some species acclimate to recurrent bleaching, others do not, emphasizing the species-specific nature of recovery dynamics^18,21^. So, while some corals can recover if the heat stress is not too severe or prolonged^17^, there remains a notable gap in understanding the short-term response of corals to such events.

The mismatch between the frequency of bleaching events and the rate of coral recovery poses a significant threat to coral survival^20^. Understanding the factors influencing recovery rates is increasingly critical in the face of climate change-induced alterations to disturbance regimes^23^. As coral reefs face increasing threats from climate change-induced events like mass bleaching, urgent actions are needed to strengthen their resilience. The international coral reef community recognizes three key pillars for coral reef conservation: (1) reducing global climate change threats, (2) enhancing reef resilience and protection through local measures, and (3) investing in restoration science^24^. Understanding the antagonistic interactions within coral reef ecosystems is essential for achieving these objectives.

Whilst the need for solutions to enhance coral resilience has been acknowledged and addressed using diverse approaches, e.g., reshaping corals’ microbial communities via the use of probiotics^25–27^, we here go beyond by investigating aids of multiple candidates to assist coral recovery. We identified four promising candidates—hydrogen, phosphate, ammonium, and probiotics—based on their documented positive effects on coral physiology under heat stress in previous studies (see Supplementary Material Table S1). Hydrogen (H_2_), exhibits properties that suggest it may serve as an effective antagonist^28^. Studies suggest that H_2_ can reduce nitrogen fixation^29,30^ and act as a strong antioxidant, thereby reducing the amount of reactive oxygen species^31–33^. Phosphate (PO_4_) may increase photosynthetic capacities along with carbon translocation in corals, and stabilizes thylakoid membranes during thermal stress^34,35^. Ammonium (NH_4_) supplementation has been associated with increased photoprotective pigments, decreased release of reactive oxygen species (ROS), and enhanced total antioxidant capacity in corals^34,36,37^. Moreover, alleviating corals from nitrogen limitation through NH_4_ supplementation increases algal cell densities and chlorophyll concentrations^38,39^. While higher symbiont densities may support post-stress recovery by increasing photosynthetic potential, they have also been linked to greater bleaching susceptibility due to increased oxidative stress under heat exposure^40^. Hence, the timing and dosage of NH₄⁺ supplementation are likely critical to avoid unintended negative effects and to maximize potential benefits. Finally, probiotics, beneficial microorganisms for corals (hereafter BMC) that have been isolated from healthy corals and screened for their beneficial traits^26^, have demonstrated promising results in promoting coral growth but also mitigating the effects of pollution, bleaching, or disease^41–50,51^.

These diverse stress antagonists offer potential avenues for boosting coral recovery from the increasing threats of heat stress induced by climate change. Ultimately, the resilience of entire ecosystems relies on the ability of foundation species like corals to recover from stressors, highlighting the pressing need for research into stress antagonists that could expedite coral recovery processes^23^. By conducting short-term experiments with the hard-coral species *Acropora* spp. and *Pocillopora favosa*, we aim to answer how various stress antagonists affect the recovery process of hard-coral species after a natural exposure to heat stress. Focusing on their physiological parameters, we aim to provide valuable insights for conservation strategies in the face of increasing heat exposure.

## Material and methods

### Experimental design

**Fig. 1:**
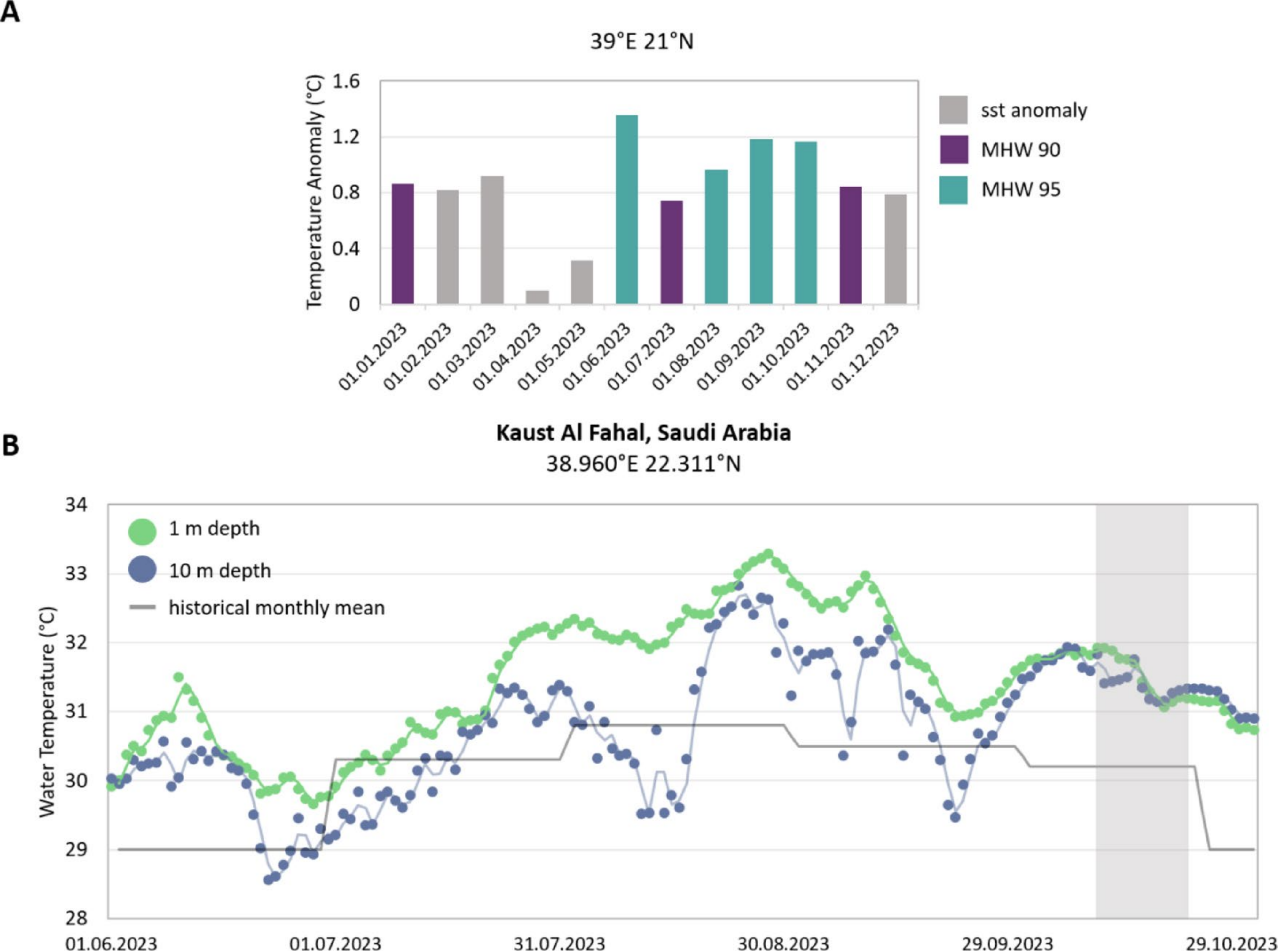
Temperature anomalies and water temperature in the study region. Panel (**A**) shows the temperature anomalies measured from satellite data of NOAA (https://psl.noaa.gov/marine-heatwaves/, accessed on 08.04.2024^54^) for the area of the sampled reef during the year 2023. Colors indicate if the months are classified as marine heatwaves (MHW) with thresholds calculated as the 90th (MHW 90) or 95th (MHW 95) percentile of observed sea surface temperature anomalies in a 3-month window. Panel (**B**) shows the daily mean water temperatures measured et al. Fahal Reef in 1 m and 10 m depths alongside the historical monthly mean (https://aqualink.org/sites/3376, accessed on 08.04.2024). The gray box indicates the field samplings between October 11th and 24th.

In 2023 the Central Red Sea experienced a bleaching event due to a long-lasting MHW, starting in June and holding on until November (Fig. 1A). A MHW is defined as a period of time in which the water temperatures are above the 90th percentile value for that location based on historical measurements^52^. Over the timespan from the beginning of June until the sampling in October, corals experienced approximately an accumulated 17 degree heating weeks (DHWs, as defined in Liu et al.^53^, Supplementary Figure S1) due to these temperature anomalies of the MHW^54^. The corals in the field experienced water temperatures of up to 33.9 °C in late August and mid-September (Fig. 1B)^55^, leading to widespread bleaching and mortality of corals of the sampling site at Al Fahal Reef (22°18′19.1′′N, 38°57′55.0′′E), but also other reefs in the area (Ref.^55^ and personal observation). At the beginning of October, water temperatures started to drop, and, soon after, surviving coral colonies started to slowly recover (personal observation).

**Fig. 2:**
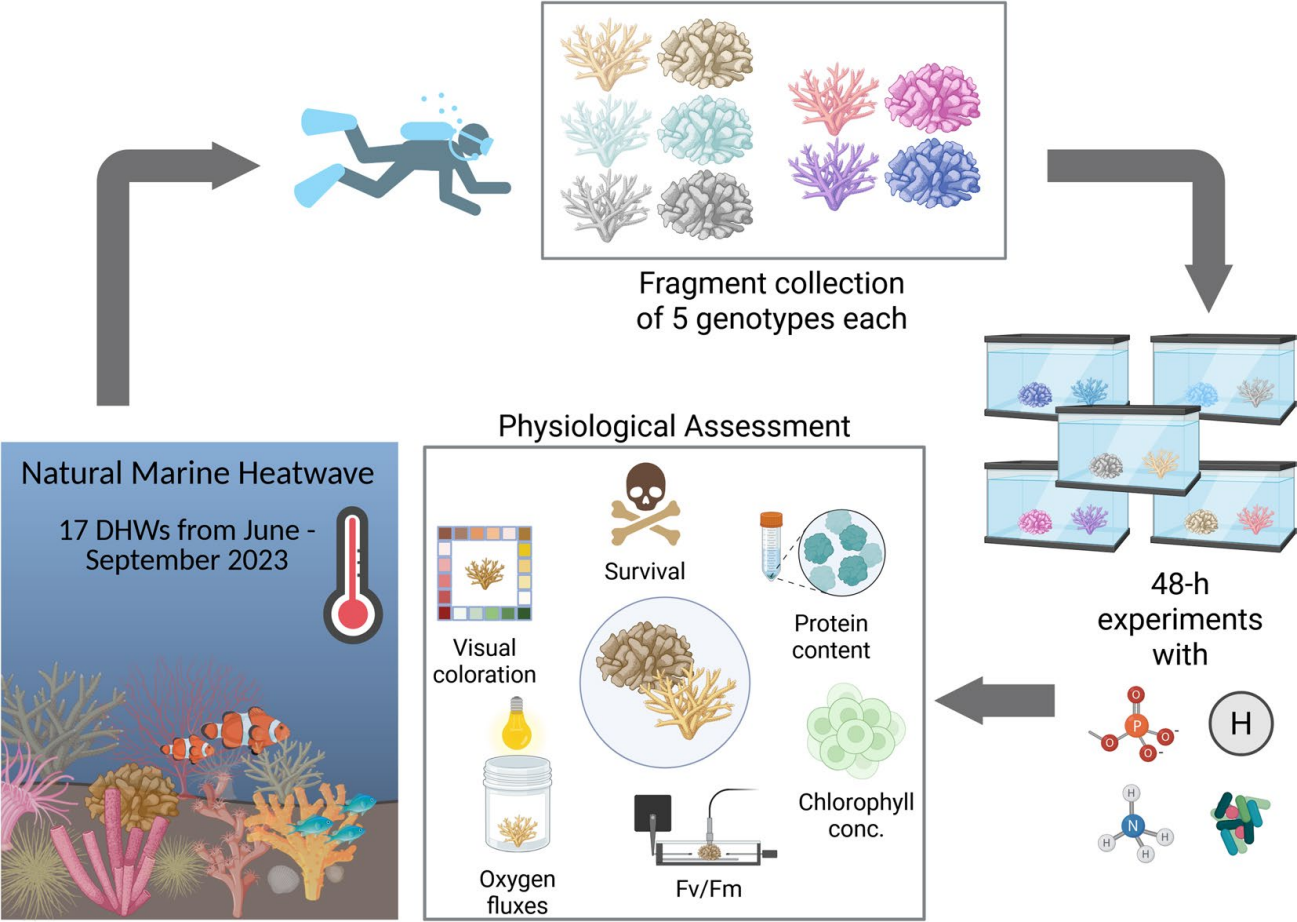
Experimental design of the study. After a long-lasting marine heat wave with an accumulated 17 DHWs, fragments of five genotypes of *Acropora* spp. and *P. favosa* were collected from paled colonies in the field, transferred into small mesocosm tanks and exposed to either of five treatments at 30 °C for 48 h before undergoing a physiological assessment to determine their recovery. Infographics were created with BioRender.

To assess the short-term effect of different stress antagonists on the recovery potential of hard corals after natural heat exposure to this MHW we collected two hard coral species, *Acropora* spp. and the recently re-classified *Pocillopora favosa*^56^, commonly found in the Red Sea (Fig. 2). For the *Acropora* fragments, we visually targeted specimens resembling *Acropora cf. hemprichii*. However, due to the genus’ pronounced morphological variability and unresolved taxonomic distinctions, we conservatively refer to these samples as *Acropora spp.* throughout this study. Fragments (n = 5 per treatment) were sampled at the “Red Sea Research Center Coral Probiotic Village” in Al Fahal Reef (22°18′19.1′′N, 38°57′55.0′′E), located about 15 km off-shore from King Abdullah University of Science and Technology (KAUST), Thuwal, in the Saudi Arabian coast of the Red Sea. Sampling was conducted while SCUBA diving, using pliers (Pliers, ACE) to cut coral fragments about 2–4 cm in length from five independent colonies per species at approximately 10 m depth, so that each treatment was represented by one fragment per colony (five fragments in total). This ensured that all treatments and subsequent physiological analyses included the same five biological replicates (colonies). All colonies, upon collection, were paled compared to their status before the MHW. Colonies were marked to allow resampling of the same colonies throughout the experiment duration. Due to limited availability of aquaria tanks, LED aquarium lights, and general laboratory space for the setup, we conducted each treatment sequentially: completing one full experimental run (i.e., exposure to one of the overall four treatments) before initiating the next. All runs were completed within a two-week period in October 2023, conducted in the following order of treatments: phosphate, probiotics, control, ammonium, and hydrogen.

On each sampling day, two fragments were cut from each of the five colonies (under protocol number 22IBEC003_V2 approved by the Office of Research Compliance—Institutional Biosafety and Bioethics Committee at KAUST) and placed in black sampling bags filled with seawater along with 80 L of seawater in plastic buckets. For the hydrogen experiment, one of the *Acropora* spp. samples got lost, reducing the replication to four genotypes in that run. Fragments were then transported to the laboratory in a plastic box (Cooler Rubbermaid 25 × 36 cm 22 L) filled with seawater from the sampling site, to shield them from high irradiation and temperatures on board during boat transfer. After arrival, all fragments were photographed for visual coloration, then, from each colony, one fragment was frozen immediately and placed in the − 80 °C freezer for baseline physiological analyses (start-point sample), and the other fragment was allocated to the experimental tanks, placed into a 3D printed holder and transferred to its respective experimental tank (end-point sample).

Experimental tanks were small, closed glass aquaria filled with 10 L of seawater collected from the reef. In total 5 small mesocosm tanks were used, each holding one fragment from a different colony of *Acropora* spp. and one fragment from a different colony of *P. favosa*, to avoid tank effects. The experimental tanks were equipped with an aquarium pump (Barst Submersible Pump, JR-250B) and a heater (Schego heater titanium tube IP68, 200 W 250 mm × 25 mm) connected to a thermostat (Inkbird temperature controller ITC-308) while natural lighting was simulated with a LED aquarium light (Galaxyhydro) replicating a 12 h light/dark-cycle. Routine monitoring during the 48-h experimental runs focused on temperature, salinity, and light intensity to ensure consistent tank conditions (tank parameters: PAR ~ 200 µmol m^−2^ s^−1^, salinity 39.6 ± 0.3 ‰, temperature 30.0 ± 0.4 °C). To simulate realistic post-heatwave conditions, all experimental tanks, including the control, were maintained at around 30.0 °C, corresponding to the historical average sea surface temperature for October and November in the region (30.5 and 30.2 °C, respectively)^57^. Our control treatment therefore represents a no-intervention baseline under ambient thermal conditions, allowing us to assess the added benefit of stress antagonists during the early recovery phase. Salinity was adjusted three times a day, in the mornings when the light was turned on, midday, and in the evening when the lights were turned off, to avoid rising salinity values in the closed aquaria system. Nutrient concentrations were not continuously measured due to the short duration of the experiment runs.

On each sampling day, an experiment with a different treatment was started, with n = 5 fragments per coral species. These five fragments always originated from the same five colonies (one fragment per colony per treatment), and all physiological analyses were performed on this same set of replicates. Treatments consisted of (1) “control” with no addition of anything, (2) “hydrogen” (H_2_) at a concentration of 0.3–0.5 ppm, equivalent to 150–250 µM, by exchanging 10% of tank water with hydrogen-enriched distilled water, supplied with marine sea salt (Red Sea salt, Red Sea) using a hydrogen water generator (Hydrogen-rich Water Cup, ABS-FQ-02, Aukewel), (3) “phosphate” (PO_4_) at a concentration of 2 µM (Sigma Aldrich, Na_2_HPO_4_ × 2 H_2_O, CAS 10028-24-7), (4) “ammonium” (NH_4_) at a concentration of 3 µM (Sigma Aldrich, NH_4_Cl, CAS: 12125–02-9), and (4) a “probiotic” treatment using tailor-made BMCs. We used two custom BMCs provided by the Marine Microbiomes Lab at the King Abdullah University of Science and Technology (KAUST). BMC I, designed for *P. favosa*, contains six bacterial strains (*Halomonas* sp., *Suctlifiella* sp., two strains of *Pseudoalteromonas* sp., and two strains of *Cobetia* sp.)^58,59^, while BMC II, designed for *A. cf. hemprichii* contains four bacterial strains (*Halomonas* sp., *Cobetia* sp., and two strains of *Pseudoalteromonas* sp.)^59^.

### Oxygen fluxes

For oxygen flux measurements, fragments were placed into a 3D-printed holder and placed into an incubation jar without being exposed to air. Jars were closed airtight for the duration of both the light and dark incubations and placed in a water bath set to the respective treatment temperature and on magnetic stirrer plates (Hanna instruments, Magnetic stirrer HI 200 M) with a magnetic stirrer in each jar to ensure constant water mixing. At the beginning and end of each incubation the oxygen concentration in the water was measured using an Oxygen probe (Mettler Toledo SevenGo Duo Pro with InLab OptiOx). Both light and dark incubations were around 1.5 h long, with light incubations using an LED Aquarium Light with an intensity of around 200 µmol m^−2^ s^−1^. After the dark incubation fragments underwent PAM measurements. Oxygen flux data, net photosynthesis (Pnet) and respiration (R) rates, was calculated by subtracting start and end oxygen concentrations of the light and dark incubations, respectively. Rates were then normalized by the surface area of each fragment (SA), total volume of the incubation jar (V), total incubation time (h) and the background oxygen fluxes (blank) as follows:

$$Pnet or R = \left( {oxy/h - blank/h} \right) * V/SA$$

Using Pnet and R, the gross photosynthesis (Pgross) was then calculated according to following formula:

$$Pgross = Pnet + \left| R \right|$$

### PAM measurements

Photosynthetic efficiency was measured directly at the start of the experiment, after 12 h, 24 h and after the dark incubation after 48 h using a Diving PAM (DIVING-PAM-II/R, Walz GmbH) after the corals were dark-adapted for > 30 min. For each fragment, the dark level fluorescence yield (*F0*) and the maximum fluorescence yield (*Fm*) were measured and the optimal PSII quantum yield (*Fv/Fm*), hereafter named photosynthetic efficiency, was calculated for a random point placed on the coral fragment. In addition, a rapid light curve (RLC) was generated for a different point through an exposure of ten light-series with increasing irradiance from 0 to 1455 μmol photons m^−2^ s^−1^. Between the twelve light-series, the fragments were illuminated with the respective actinic light for 10 s, each ended by a saturation pulse.

After the PAM measurements, fragments were photographed for visual coloration and survival assessment, and subsequently stored in the − 80 °C freezer until further analysis.

### Visual coloration and survival

Visual coloration was assessed by taking pictures of each fragment against a white background using a mobile phone camera (iPhone 7) before the start of the experiment and at the end of the 48 h stress antagonist run. Pictures were analyzed following the protocol of Mclachlan & Grottoli^60^ using the software ImageJ taking ten separate gray values per fragment, standardizing them against the white background, and calculating the mean intensity gray (MIG) values. MIG values were inverted, so that a lower value, or a reduction, indicates paling/bleaching of the fragment, while higher values correlate with darkening of the fragment.

Survival was determined by less than 75% tissue loss of the fragments. If fragments showed > 75% tissue loss this was defined as mortality, similarly to a study by Casey et al.^61^

### Airbrushing and surface area determination

Hard coral fragments were defrosted on ice for 1 h before airbrushing the tissue off the skeleton using 5 ml of micro filtered seawater and an airbrush gun. Tissue slurry was collected in a falcon tube (VWR 15 ml Centrifuge Tube, Cat. No. 525-1084) and then transferred into 3 different 1.5 ml Eppendorf tubes (Fisher Scientific, Cat. No.: 05-408-137) and frozen again at − 80 °C until further physiological analyses.

Surface area was determined by building a 3D model using the software Agisoft Metashape (Version 2.1.0, Professional) from 50 pictures taken of each fragment. Models were then analyzed using MeshLab (Version 2023.12).

### Chlorophyll content

To measure total pigment content, slurries were removed from the freezer and placed on ice for thawing. All steps of measurement preparation were conducted in the dark under red light. Slurries were homogenized by vortexing them (VWR vortexer mini, Cat. No.: 58816-123). Afterward, 500 µl of samples were aspirated into a 2 ml Eppendorf tube (Fisher Scientific, Cat. No.: 05-408-146) and centrifuged for 5 min (4 °C, 5000 rpm, Eppendorf centrifuge 5424 R). Supernatant was discarded and 2 mL of 90% acetone (VWR, Acetone for HPLC, Cat. No.: 20067.320) added. Samples were sonicated in an ice water bath for 10 min (Bransonic Ultrasonic Cleaner, model: 5510E-MTH) and then placed inside a cardboard box in a fridge at 4 °C for 48 h of extraction time. After the extraction, samples were taken from the fridge, vortexed again for 2 s and then placed in a benchtop centrifuge (Cole parmer, Veron Hills, Illinois 60061) for 5 s before pipetting 200 µl of supernatant into the respective well plates in duplicates. In each 96-well plate (VWR Tissue Culture plate, European Cat. No.: 734-2327) two wells were filled with 200 µl of 90% acetone for blanks. Samples were then placed in a spectrophotometer plate reader (thermo scientific, spectrophotometer, Ref.: A51119700) and read at wavelengths of 630, 663, 664, and 750 nm wavelength. Sample absorbance values were corrected by subtracting the average absorbance of the blanks. Afterwards, chlorophyll *a* and *c2* concentrations were calculated using the equations of Jeffrey & Humphrey^62^, while accounting for the path length of the well plate by dividing the concentrations by 0.555^63^. After that values were converted from concentration to the absolute amount of extracted chlorophyll, by multiplying them with the extraction volume, and then multiplied by the dilution factor. Lastly, we standardized the absolute amount of chlorophyll *a* and *c2* to the surface area of the respective fragment.

### Protein content

Protein concentrations were determined using a Bradford assay kit (Thermo Scientific, Coomassie Protein Assay Kit, Ref.: 23,200). First, a dilution of standards was mixed using an Albumin Standard (Thermo Scientific, Albumin Standard, Ref.: 23,209) and MilliQ water for dilution. Tissue slurry was thawed on ice and then homogenized thoroughly by vortexing (VWR vortexer mini, Cat. No.: 58816-123). Depending on the sample the tissue slurry was diluted in a new 1.5 ml Eppendorf cup (Fisher Scientific, Cat. No.: 05-408-137) with MilliQ water to fall into the range of the standard curve. After homogenization, the samples were placed in a small benchtop centrifuge (Cole Parmer, Veron Hills, Illinois 60,061) for 5 s. Then, 5 µl of sample supernatant was transferred in technical triplicates in a 96-well plate (VWR Tissue Culture plate, European Cat. No.: 734-2327). Using a multichannel pipette 250 µl of Coomassie reagent was transferred into each well and properly mixed with the sample without producing bubbles. The 96-well plate was placed on a shaker (Eppendorf, MixMate) for 30 s at 500 rpm, and subsequently incubated at room temperature for 10 min. After the incubation, the plate was placed back on the shaker for 30 s at 500 rpm, and then put into a spectrophotometer plate reader (Thermo Scientific, spectrophotometer, Ref.: A51119700) to measure the absorbance of the wells at a wavelength of 595 nm. Sample absorbance was corrected by subtracting the average absorbance of the blank standard, then absorbance values more than 0.03 units away within the triplicates were discarded. Protein concentration was then calculated using the standard curve produced (using a quadratic curve fit, forced through the origin), multiplied by the respective dilution factors of tissue slurry used and additional dilutions, and, finally, standardized to surface area.

### Data analysis

To account for differences in initial values across separate experimental runs, which resulted from the logistical constraint of conducting each treatment sequentially rather than simultaneously, we calculated the percentage change from start to end point for each individual fragment. This approach was applied to all physiological parameters assessed, including visual coloration, Fv/Fm, chlorophyll *a* content, and protein concentration. By focusing on the relative change over time within each fragment, we minimized the influence of between-run variability and ensured that comparisons reflect treatment-specific effects rather than differences in baseline conditions. To further standardize the data across treatments, percentage change values were normalized using the formula

$$Value Normalized = \left( {Value Original/100} \right) + 1$$

which centers values around 1 and facilitates interpretation of increases and decreases on a comparable scale. Data analysis was then conducted in R version 4.3.3^64^ using the packages stats^64^ and emmeans^65^. All analyses were performed separately for each species. Prior to statistical testing, assumptions of normality and homogeneity of variances were evaluated using the Shapiro–Wilk and Bartlett tests, respectively.

To assess differences between treatments for each species at the end of the experiment (48 h), a one-way analysis of variance (ANOVA) was applied on the percent change data when assumptions were met. Post hoc comparisons were conducted using *emmeans* with Benjamini–Hochberg (BH) adjustment for multiple comparisons. When assumptions of normality or homoscedasticity were violated, a non-parametric Kruskal–Wallis test was used, followed by pairwise Wilcoxon rank-sum tests with BH-adjusted *p* values. To evaluate changes over time within each treatment, two-sample t-tests were applied on the raw data when assumptions of normality were met in both timepoints. When normality was violated for one or both timepoints, the non-parametric Wilcoxon rank-sum test was used. Tests were carried out separately for each treatment within each species. To allow for reliable analysis of physiological parameters, we only included fragments that were still alive at the end of the experiment. Dead fragments were excluded from the analyses of Fv/Fm, chlorophyll, oxygen fluxes, and protein content, as no tissue remained for measurement. However, the mortality data was kept to analyse treatment effects on survival. Hence, the statistics and figures of these parameters are the result of the replicate numbers given in Table 1.

**Table 1:** Number of fragments of *Acropora* spp. and *P. favosa* alive after the 48 h experimental period and used for analysis of Fv/Fm, chlorophyll, oxygen fluxes, and protein content.

Treatment	*Acropora* spp.	*Pocillopora favosa*
Control	3 of 5	2 of 5
Hydrogen	3 of 4	4 of 5
Phosphate	5 of 5	4 of 5
Ammonium	5 of 5	4 of 5
Probiotics	4 of 5	4 of 5

All results were considered statistically significant at *p* < 0.05. Non-significant results are also reported, as this study represents a novel exploratory assessment of potential stress antagonists aiding hard coral recovery. Therefore, trends that do not reach statistical significance are still included as potential indicators for future targeted testing under more robust experimental conditions.

## Results

### Control treatment

**Fig. 3:**
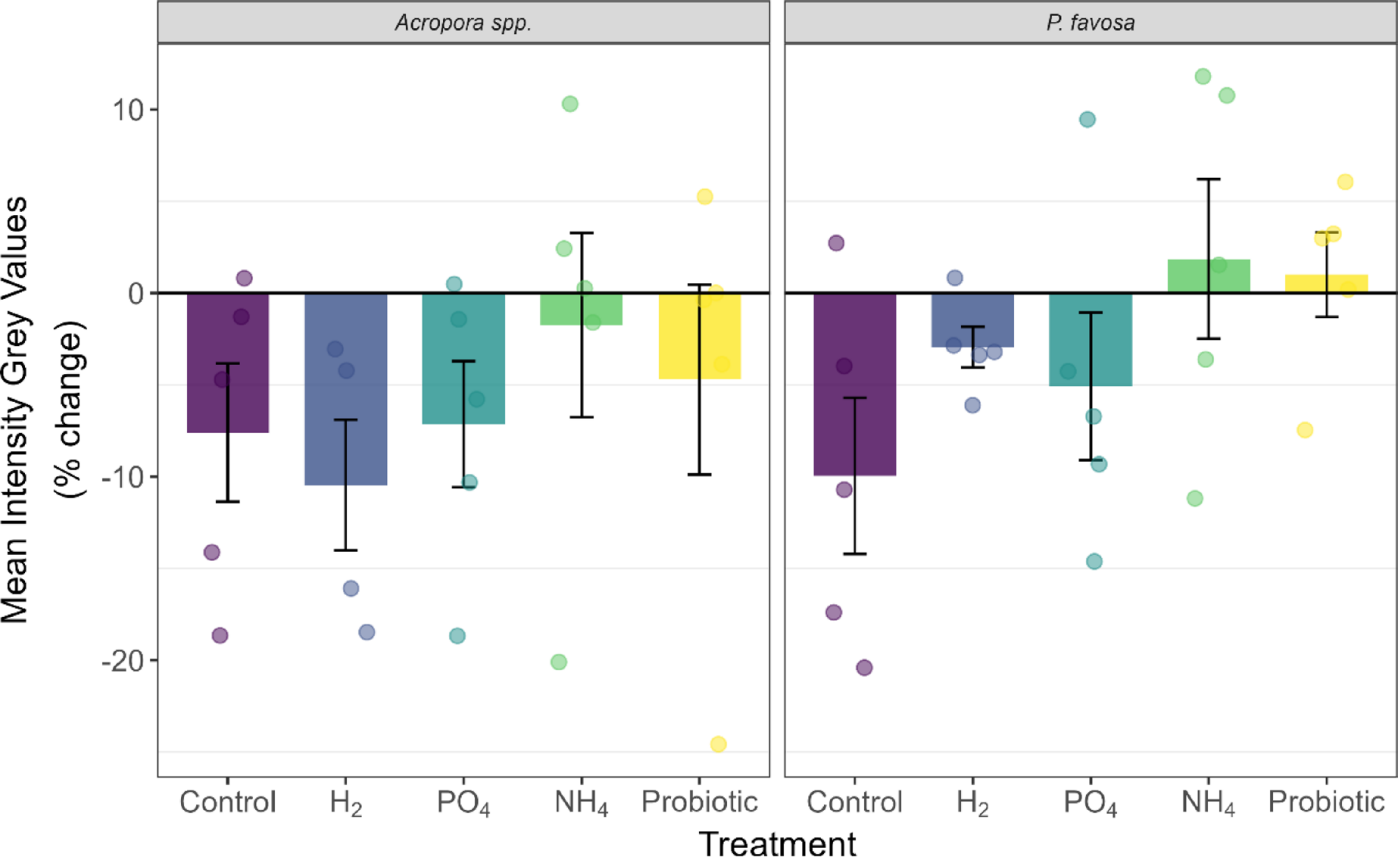
Percent change in Inverted Mean Intensity Grey Values of *Acropora* spp. and *P. favosa* over the 48 h experimental period. Barplots show the mean % change with error bars representing the standard error. Each separate measurement is indicated by a dot in the respective treatment color.

**Fig. 4:**
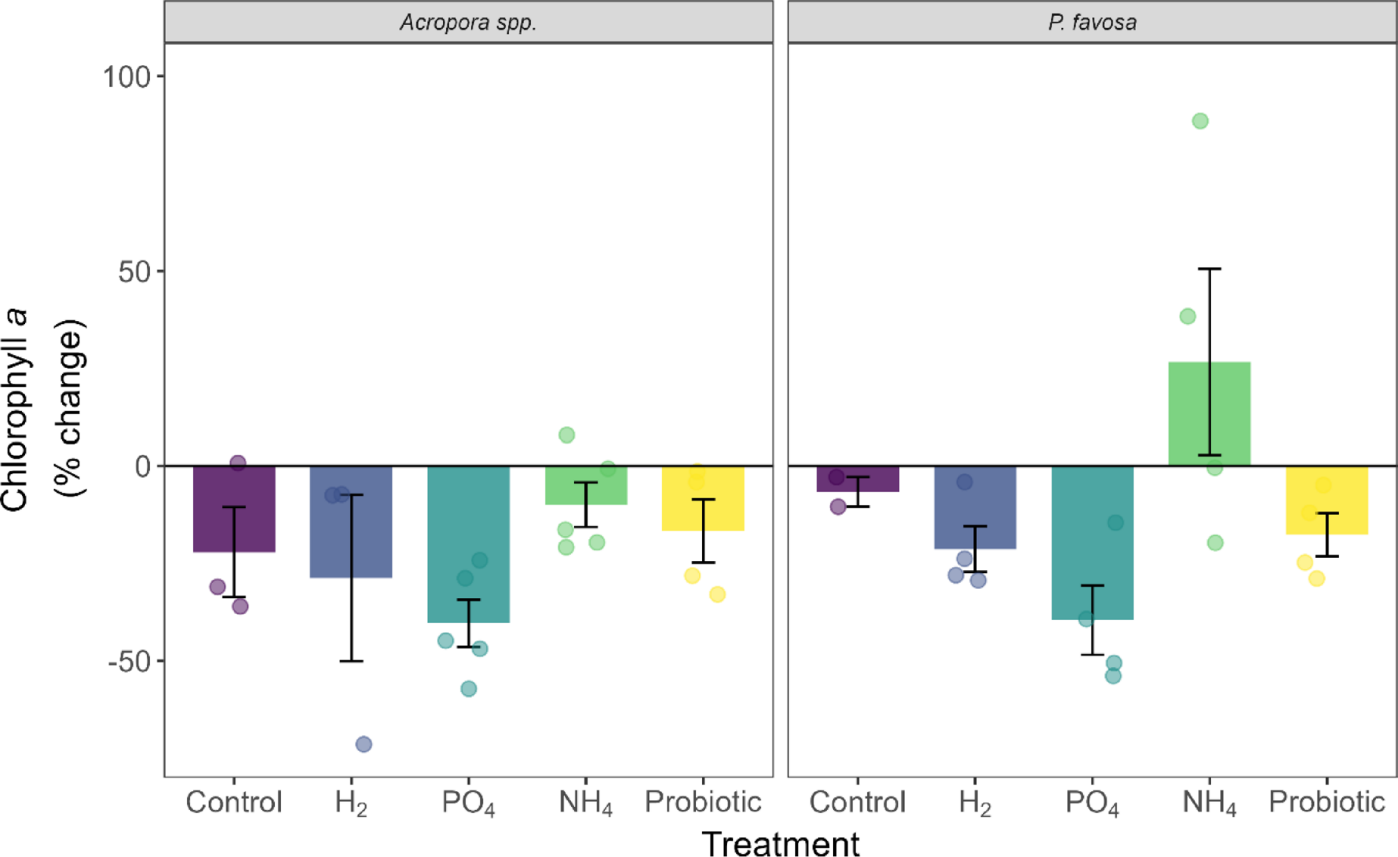
Percent change in Chlorophyll *a* concentration of *Acropora* spp. and *P. favosa* over the 48 h experimental period. Barplots show the mean % change with error bars representing the standard error. Each separate measurement is indicated by a dot in the respective treatment color.

**Fig. 5:**
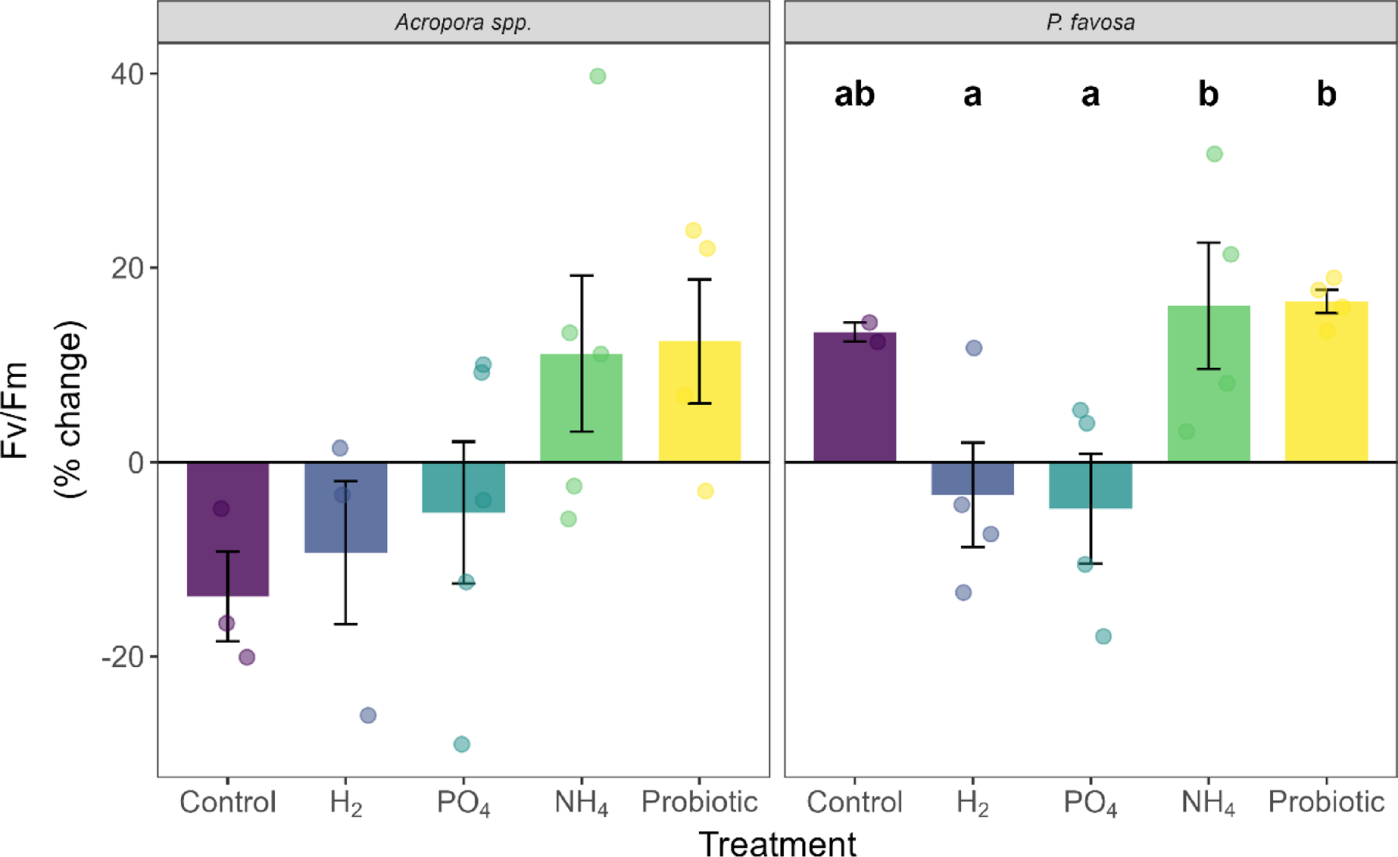
Percent change in photosynthetic efficiency (Fv/Fm) of *Acropora* spp. and *P. favosa* over the 48 h experimental period. Barplots show the mean % change with error bars representing the standard error. Each separate measurement is indicated by a dot in the respective treatment color. Different letters above the bars indicate significant differences between treatments (*p* < 0.05).

**Fig. 6:**
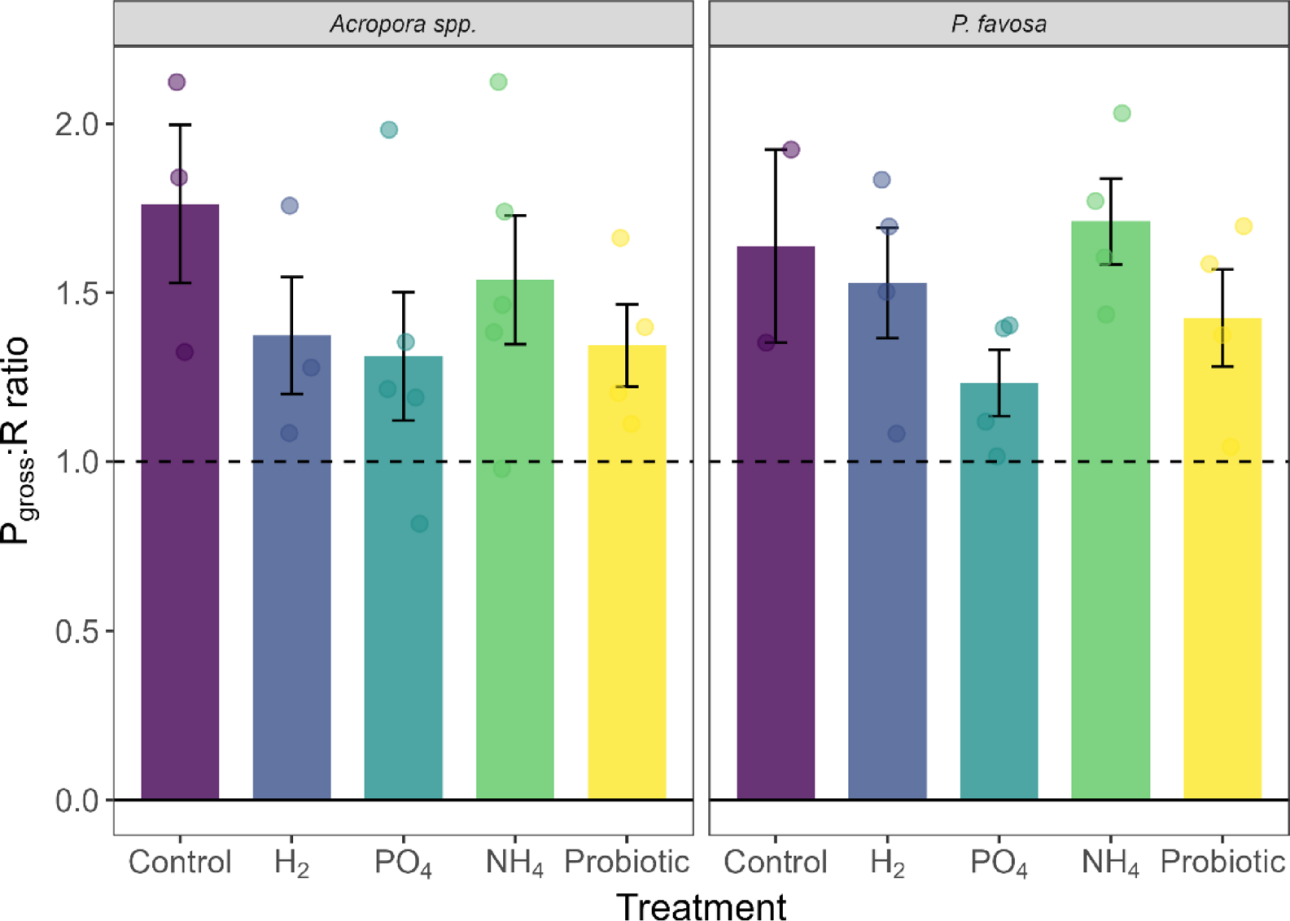
Pgross:R ratio of *Acropora* spp. and *P. favosa* over the 48 h experimental period. Barplots show the mean % change with error bars representing the standard error. Each separate measurement is indicated by a dot in the respective treatment color. The dashed line indicates a ratio of 1 with values above the dashed line representing greater energy acquisition than utilization.

At the end of the 48-h period of the experiment, a survivorship of 3 out of 5 fragments was observed for *Acropora* spp. in the negative control treatment (Table 1). A reduction of 8% in coloration (Fig. 3), 22% in chlorophyll *a* (Fig. 4), and 34% in chlorophyll c2 (Supplementary Fig. S2) were observed over the 48-h period of the experiment, yet these were not statistically significant declines. Additionally, no significant differences were observed in photosynthetic efficiency (Fig. 5) nor oxygen fluxes over time for *Acropora* spp. However, the highest P_net_ values (Supplementary Fig. S3) at 75.0 mg O_2_ m^−2^ h^−1^, and highest respiration values at − 99.3 mg O_2_ m^−2^ h^−1^ were observed, leading to a P_gross_:R ratio of 1.8 (Fig. 6), which was the highest of all treatments. Protein content did not change (Supplementary Fig. S4).

As for *P. favosa,* only 2 out of 5 fragments in the negative control tanks survived (Table 1). Over the 48-h period, their coloration was reduced by 10% (Fig. 3). Although not statistically significant this was the highest color loss among all treatments, which aligned with the decreased values in both chlorophyll *a* (Fig. 4) and c2 values (Supplementary Fig. S2) of − 7% each. Surprisingly, photosynthetic efficiency increased over time by 13% in the two fragments that survived, yet due to the low sample size this change was not significant (Fig. 5). Oxygen fluxes were similar to the ones in the other treatments with a P_gross_:R ratio of 1.6 (Fig. 6 and Supplementary Fig. S3).

### Hydrogen treatment

Compared to the control treatment, in the H_2_ treatment the survival of *Acropora* spp. was 3 out of 4 fragments (Table 1). Additionally, coloration dropped by 10% (Fig. 3), with a 29 and 18% decrease in chlorophyll *a* (Fig. 4) and c2 concentrations (Supplementary Fig. S2), respectively. Hydrogen also led to a similar decline in photosynthetic efficiency as the control, with -9%. With mean P_gross_ rates of 124.0 mg O_2_ m^−2^ h^−1^ and R rates of − 88.2 mg O_2_ m^−2^ h^−1^, the P_gross_:R ratio was 1.4 overall. However, the trends observed for coloration, chlorophyll content, and oxygen fluxes, were not statistically significant (*p* > 0.05).

For *P. favosa*, on the other hand, H_2_ doubled survival to 4 out of 5 fragments compared to the control treatment (Table 1). Coloration and photosynthetic efficiency both decreased by 3% throughout the 48 h of the experiment, yet not significantly (*p* > 0.05). Also, a non-significant loss in chlorophyll *a* and c2 by 21 and 16%, respectively, was measured. The oxygen fluxes under H_2_ enrichment were not significantly different from the other treatments and led to an overall P_gross_:R ratio of 1.5. Finally, protein content decreased by 6% under H_2_ enrichment, yet this was no significant decline.

### Phosphate treatment

For both hard corals, PO_4_ increased survival by 2 fragments compared to the control, to an overall survival of 5 out of 5 fragments in *Acropora* spp. and doubled survival to 4 out of 5 fragments in *P. favosa* (Table 1). Surprisingly, in both hard corals the decrease in chlorophyll *a* and c2 was the highest, with a significant decrease in *P. favosa* over time (*p* = 0.026 for Chl a and *p* = 0.023 for Chl c2), yet compared to the other treatments this change was not significant (*p* > 0.05; Fig. 4 and Supplementary Fig. S2). Additionally, for both hard corals respiration under PO_4_ enrichment was the lowest for *Acropora* spp. and *P. favosa* with only − 79.0 and − 84.9 mg O_2_ m^−2^ h^−1^, respectively, but not significantly different from the controls (*p* > 0.05). Together with the lowest P_gross_ rates of all treatments, the P_gross_:R ratio was 1.3 and 1.2 for *Acropora* spp. and *P. favosa* (Fig. 6).

### Ammonium treatment

Similarly to PO_4_, NH_4_ also increased survival by 2 fragments, to an overall survival of 5 out of 5 fragments in *Acropora* spp. (Table 1). While the other physiological parameters did not change significantly, trends are still visible. Looking at the coloration NH_4_ led to the least severe decline in coloration with − 2%, and chlorophyll *a* and c2 with − 10 and − 9%. Photosynthetic efficiency increased by 11% over the 48-h period (*p* = 0.142, Fig. 5). With P_gross_ and R rates being similar to the ones of other treatments, the overall P_gross_:R ratio was the second highest of all treatments with 1.5 (Fig. 6). Protein content decreased by 15% under NH_4_ enrichment (Supplementary Fig. S4).

*P. favosa* showed similar trends to *Acropora* spp. under NH_4_ enrichment with a doubling of survival to 4 out of 5 fragments (Table 1). Besides this, NH_4_ increased the coloration of fragments by 2%, most likely due to increased levels of chlorophyll *a* by 27% and chlorophyll c2 by 39% over the 48 h, though these changes were not significant. Yet, NH_4_ significantly increased photosynthetic efficiency by 16% over time (*p* = 0.026, Fig. 5), being significantly higher than in the H_2_ or PO_4_ treatment (*p* = 0.038 for both), leading to the highest measured P_gross_:R ratio of 1.7 among all treatments (Fig. 6).

### Probiotics treatment

The probiotics treatment increased the survival of *Acropora* spp. to 4 out of 5 fragments(Table 1) . While coloration and chlorophyll *a* and c2 concentrations decreased over the 48 h by 5, 17, and 9%, respectively, these changes were not significant. Nevertheless, photosynthetic efficiency significantly increased by 12% over time (*p* = 0.046, Fig. 5). Overall *Acropora* spp. fragments showed a P_gross_:R ratio of 1.3.

Applying the probiotic treatment on *P. favosa* led to doubling their survival to an overall 4 out of 5 fragments (Table 1). Chlorophyll *a* and c2 concentrations showed declines of 18 and 19%, respectively, yet these were not statistically significant (*p* > 0.05). Additionally, photosynthetic efficiency significantly increased by 17% over time (*p* = 0.001, Fig. 5), being significantly higher than in the H_2_ or PO_4_ treatment (*p* = 0.038 for both). Interestingly, the probiotics treatment led to the highest R rate with − 121.6 mg O_2_ m^−2^ h^−1^, and the highest P_gross_ rates with − 174.5 mg O_2_ m^−2^ h^−1^ among all treatments (Supplementary Fig. S3), leading to an overall P_gross_:R ratio of 1.4 (Fig. 6).

## Discussion

To our knowledge, this study is the first to compare the effects of different stress antagonists on short-term coral recovery after a MHW. Through this research, we aimed to improve our understanding of recovery from heat stress, under the influence of different potential stress antagonists, therefore providing alternative tools for customized medicine for corals^66^. As all stress antagonists tested showed higher survival rates of fragments than the control, with *P. favosa* survival even doubling in all treatments, we encourage complementary use of these findings based on the needs and available logistics at a given site.

### Effects of hydrogen

In our control treatment, corals showed clear signs of stress during the 48-h experiment, with the lowest overall survival and coloration. Under H_2_ enrichment, we observed an increase in survival for both *Acropora* spp. and *P. favosa* , with survival rates increasing to 3 out of 4 fragments for *Acropora* spp. and doubling of survival to 4 out of 5 fragments for *P. favosa* fragments. H_2_ is recognized as a potent antioxidant in other organisms such as mice, rats, and humans^31–33^. According to the oxidative theory of coral bleaching, under heat stress, reactive oxygen species (ROS) are produced as a result of the impaired photosynthetic machinery in the symbiont and escape into the host cell, overwhelming its antioxidant system and causing damage to the host tissue^67,68^. Whether the corals can recover or if they die depends on the extent of oxidative damage from ROS. Minor ROS-induced damage can potentially be repaired, allowing the coral to recover, while irreparable damage to essential cellular structures can lead to coral mortality^67^. Hence, the antioxidant properties of H_2_ can play a vital role in aiding coral recovery after a MHW.

While there were slight differences in the oxygen fluxes of fragments in the control and the H_2_ enrichment, none of the differences was significant. The slightly reduced primary productivity in *Acropora* spp. fragments could be a consequence of a possible reduced nitrogen fixation induced by H_2_, which then reduced nitrogen availability—a nutrient known to affect photosynthetic rates^69,70^. In addition, our findings of a slightly reduced coloration and chlorophyll content might also be indicative of a beginning H_2_-induced nitrogen limitation, with H2 being a competitive inhibitor of the nitrogen fixation in diazotrophs^29,30,71^, as nitrogen limitation can hinder the growth of endosymbiotic algae^72,73^. This might further explain the observed reduced photosynthetic rates. Despite this, the mean P_gross_:R ratio remained above 1 for each treatment, meaning that they were acquiring more energy through autotrophy than they were using^74^. Furthermore, we want to highlight that corals might have experienced a post-heat stress disorder (PHSD), a term introduced by Santoro et al.^46^. PHSD is characterized by cellular, immune, and metabolic consequences of heat stress that corals experience for days up to weeks after the end of a heat-stress period. These consequences include, for example, increased inflammation and tissue damage, apoptosis, and cellular reconstruction of membranes and the cytoskeleton. Some of these effects seem to be triggered even before visual signs of bleaching in coral fragments, as processes like altered nutrient cycling during heat stress can drive the breakdown of the coral–algal symbiosis, leading to bleaching and coral mortality later on^75^.

Overall, while our study highlights the complexity of the effects of H_2_ on coral physiology, further research is warranted to unravel its role, both at a more mechanistic level, looking at the nitrogen cycle in the coral holobiont, and at the level of the reef community and the effects on other surrounding organisms.

### Effects of phosphate

The improvement in survival, reaching 5 out of 5 fragments for *Acropora* spp. and a doubling in survival to 4 out of 5 fragments for *P. favosa* compared to controls, underlines the potential effectiveness of PO_4_ as a stress antagonist in short-term coral recovery efforts. Previous studies highlight the detrimental effects of PO_4_ starvation on the coral-algal symbiosis, including reduced bleaching thresholds and impaired algal photosynthesis^35,76^. Furthermore, Rädecker et al.^75^ proposed a feedback loop in which heat stress increases the metabolic energy demand of corals, which they compensate for by catabolically degrading amino acids, or by digestion of symbiont cells^77^. This degradation of amino acids leads to a shift from net uptake to release of ammonium by the coral holobiont, which then promotes the proliferation of the symbiotic algae instead of providing photosynthates to the coral host. This creates a feedback loop that gradually alters the state of the nutrient cycle between the coral host and the algal symbiont^75^, potentially exacerbating PO_4_ limitation. In this context, PO_4_ enrichment may mitigate stress-induced nutrient imbalances and thereby enhance coral survival, as shown in our study.

Additionally, Ezzat et al.^34^ demonstrated that PO_4_ supplementation, coupled with a reduction in the nitrogen to phosphorus (N:P) ratio, prevented coral bleaching and improved photosynthetic performance under heat stress. This suggests that PO_4_ enrichment not only maintains coral health, but also enhances physiological processes critical for thermal tolerance. However, while our study highlights the potential benefits of PO_4_ enrichment for short-term coral recovery, caution must be exercised in its application. We suggest that PO_4_ enrichment may be more effective as a preventative measure rather than as a post-heat stress intervention. Proactive enrichment of PO_4_ levels before or during heat events could help to maintain nutrient balance and alleviate stress-induced nutrient limitation, thereby pre-empting the negative feedback loop described by Rädecker et al.^75^.

In conclusion, our results highlight the importance of considering nutrient dynamics, particularly PO_4_ availability, in coral conservation strategies. PO_4_ enrichment shows promise as a means to enhance coral resilience to heat stress, offering potential avenues for targeted interventions in coral reef management.

### Effects of ammonium

Our study shows that NH_4_ enrichment improves short-term survival of both *Acropora* spp. and *P. favosa*, with a remarkable increase to 5 out of 5 fragments surviving in *Acropora* spp. and a doubling to 4 out of 5 fragments surviving in *P. favosa* fragments. Furthermore, fragments exposed to enriched NH_4_ conditions exhibited the highest P_gross_:R ratios, increased photosynthetic efficiency, chlorophyll concentrations and coloration values compared to other treatments. This observation is consistent with previous studies which reported increased algal densities and chlorophyll concentrations in hard corals exposed to 3–20 µM NH_4_ enrichment for periods ranging from two to five weeks^37–39,78^. The observed positive effects of NH_4_ enrichment on coral recovery can be attributed to several factors. First, NH_4_ may release algal symbionts from their nutrient-limited state, allowing them to grow faster compared to other treatments^39,78^. In addition, NH_4_ enrichment facilitates molecular repair through increased nucleic acid synthesis and protein turnover in plants^79^.

Not only NH_4_ enrichment alone can show positive effects, but also under heat stress NH_4_ enrichment proves to be beneficial. Béraud et al.^36^ found that under heat stress, corals exposed to NH_4_ enrichment had higher chlorophyll concentrations and photosynthesis rates than the heated control. This aligns with the trends observed in our *P. favosa* fragments having increased chlorophyll levels and photosynthetic efficiency. Béraud et al.^36^ argued that this may be due to an increase in photoprotective pigments in the coral tissue, which contributed to membrane protection, allowing them to maintain their photosynthesis rates compared to controls. Secondly, Fernandes de Barros Marangoni et al.^37^ also showed increased algal densities and chlorophyll concentrations together with reduced ROS concentrations under NH_4_ enrichment compared to the heated controls. In their study, NH_4_ enrichment was found to alter the deleterious effects of thermal stress by favoring the oxidative status and energy metabolism of the coral holobiont, probably due to increased carbon acquisition and translocation to the host compared to control conditions. In addition, they argued that maintaining normal algal densities may favor homeostasis under thermal stress. The proposed mechanisms of both studies could explain our overall results of higher survival, chlorophyll, and coloration values in fragments held under NH4 enrichment. NH_4_ enrichment might have influenced the amount of photoprotective pigments and reduced ROS concentrations, leading to an overall lower PHSD in those fragments over 48 h.

While previous studies showed the significant positive effects of NH_4_ enrichment on coral physiology under thermal stress, and our study further supports this through increased survival and chlorophyll concentrations, the effect of NH_4_ is still debated. Heat stress has also shown to trigger a shift from net uptake to strong net release of NH_4_ from the holobiont, mainly as a result of increased production of NH_4_ through amino acid catabolism of the coral host, which caused the expulsion of the algal symbiont (i.e., coral bleaching)^75^. In our study we did not find a strong net release of NH_4_, on the contrary the corals were taking up the NH_4_ as the concentrations measured in the water samples decreased from 2 µM at the beginning to 1.2 µM after 24 h and to 0.6 µM after 48 h. This uptake of NH_4_ in our study could probably be a result of the corals being in a post-bleaching phase at ambient temperatures instead of currently experiencing high temperatures.

Overall, studies showed that the effect of nitrogen can be beneficial^36,37,80^ or harmful^35,70,81–84^, depending on the specific concentrations, environmental conditions, and nitrogen sources. Hence, the use of NH_4_ enrichment should be evaluated in the light of current concentrations in the reef and used as a means to “correct” for rates considered too low, but not exceeding optimal concentrations. While our study provides valuable insights into the short-term benefits of NH_4_ enrichment on coral recovery, further research is also warranted to elucidate its long-term effects. Previously conducted studies in the central Red Sea with in-situ eutrophication of increasing dissolved inorganic nitrogen levels up to 1.3 µM, of which most was nitrate, showed benefits for turf algae proliferation^85^ as well as heterogeneous effects on algae- and coral-dominated reef communities^86^, and an indication that microbial N cycling might not relief but rather exacerbate eutrophication in coral reefs by increased dinitrogen fixation^87^. A follow-up experiment over a longer period of time with mainly increased NH_4_ could determine whether there is a critical threshold beyond which NH_4_ enrichment may reverse its beneficial effects and destabilize the symbiosis again. Also, investigating the effect of such NH_4_ concentrations on other benthic organisms that are in competition with hard corals, such as turf algae or soft corals, is necessary to establish a well-rounded treatment that is helpful for corals, and not accidentally proliferating algal growth^85^. Nevertheless, the results underline the potential of NH_4_ as a valuable stress antagonist for coral recovery efforts and emphasize the importance of considering nutrient dynamics in conservation strategies aimed at optimizing its use to mitigate the effects of climate change on coral reefs.

### Effects of probiotics

The use of tailor-made probiotics to enhance the short-term recovery of hard corals represents a promising avenue in coral conservation efforts. Probiotics have emerged as a rapid (days to weeks) strategy for corals to adapt to changing environmental conditions, as opposed to mutation and selection, which can take many years^26^. The selection of bacterial strains for our probiotic treatments was based on their beneficial properties, including mitigation of reactive oxygen species and nitrogen cycling, with the aim of improving coral well-being and resilience^26,88^. These probiotics act as protective agents for the coral holobiont, restoring a balanced relationship between the coral host and its associated microbiota, thereby enhancing coral health and survival that may also have a beneficial effect on the entire ecosystem^89^.

In our study, we observed improvements in survival rates for both *Acropora* spp. and *P. favosa* following probiotic treatment, with survival increasing to 4 out of 5 fragments for both *Acropora* spp. and *P. favosa* compared to control treatments. Our results are consistent with previous studies showing increased survival of corals exposed to stressors such as temperature increases^46^. In addition, we observed less bleaching in the probiotic treatment, which is consistent with previous research indicating reduced bleaching in corals treated with probiotics under temperature stress^45^. In their study, Rosado et al.^42^ demonstrated that it is possible to manage the coral microbiome in a controlled aquarium environment, and that this stewardship can influence coral health. They hypothesized that probiotics may have promoted shifts in the coral microbiome, excluding pathogens and allowing the establishment of other beneficial components, indirectly making corals less susceptible to bacterial pathogens.

Interestingly, our probiotic treatment led to the highest respiration rates measured in *P. favosa* fragments in our study. This could be attributed to the influence of probiotics on anaerobic respiration processes within the coral microbiome, potentially contributing to increased metabolic activity^27^, or even the microbes in the treatment itself. The photosynthetic efficiency values observed in our study showed a notable increase for both *Acropora* spp. and *P. favosa*, increasing by 12% and 17%, respectively. In previous studies, probiotic treatments maintained photosynthetic efficiency or also slightly increased values compared to heated controls^41,45,46^. As the amount of bleaching and reduction in chlorophyll content was less pronounced in the probiotics treatment, this suggests that the observed increase in photosynthetic efficiency might be due to mitigation of bleaching through BMCs. These results highlight the potential role of probiotics in maintaining or enhancing key physiological processes essential for coral health and resilience.

In summary, our study highlights the importance of understanding the direct and indirect pathways by which probiotics improve coral health, and provides new insights into the beneficial effects of probiotics on short-term coral recovery. Previous research has shown that increased stress tolerance in corals exposed to probiotics coincides with changes in gene expression, immune response and metabolism within the coral holobiont during recovery periods following thermal stress^46^. By promoting shifts in the coral microbiome and enhancing metabolic activity, probiotics offer a promising strategy for enhancing coral resilience to environmental stressors. However, further research is needed to elucidate the underlying mechanisms and long-term effects of probiotics on coral health and ecosystem dynamics, particularly in in-situ experiments.

### Ecological implications and outlook

Coral reefs worldwide are facing the combined effects of local stressors and climate change^4,5^. Addressing greenhouse gas emissions remains critical, but proactive interventions are needed^66^. Traditional reef management practices have demonstrated success, but active interventions are increasingly being recognised^90,91^. These include practices ranging from traditional coral propagation to new tools such as assisted evolution and microbiome manipulation^25,66,92,93^. While risk assessment of such tools is essential, innovation is urgently needed to increase reef resilience and recovery^66^.

In our study, both *Acropora* spp. and *P. favosa* showed an increased survival across all stress antagonists tested, with *P. favosa* fragments doubling the survival, suggesting a general potential for these interventions to also improve coral recovery and not just mitigate heat stress^34,36,45^. Translating such laboratory results to field applications is difficult. There are currently no reef-wide applications to provide antioxidants, nutrients or probiotics to entire reef ecosystems, but NASEM (2019) suggests using the expertise and methods of the agricultural sector to find solutions. Additionally, products are emerging that offer a potential solution to release stock solutions of nutrients or probiotics into the reef using different delivery solutions^88^ and even automated delivery systems. This could help to administer a desired antagonist to the reef over an extended period of time, and also remotely as soon as bleaching warnings occur, by installing these systems in the reef and connecting them remotely. While large-scale reef-wide application still remains a major challenge, the tested antagonists may be more immediately useful for targeted efforts such as protecting high-value reef sections, assisting transplanted nursery corals, or aiding in the recovery of threatened species in controlled environments, supporting survival and recovery. Additionally, in the future, broader nutrient manipulation may be possible with wastewater treatment plants serving as delivery systems into coastal reef areas.

Understanding optimal antagonist concentrations and their broader ecosystem impacts is critical, as, for example, skewed nutrient ratios can make corals even more susceptible to heat stress^35,94^ or support unwanted algal proliferation^85^. Concentrations should therefore always be calculated site-specifically, with concentrations kept to the minimum required to achieve a beneficial effect. As probiotics have recently been shown to have no negative effects on the surrounding environment^58^ while promoting coral health^45,46^, this makes them a particularly valuable candidate for further in-situ studies. Using our new knowledge on stress antagonists, even more tailored coral medicine could be developed, based on the specific needs at each reef^66^. It may even be interesting to combine different antagonists, but their interactive effects should first be studied in laboratory experiments to ensure that there are no negative additive or even synergistic effects^95^. By bridging laboratory investigations with field-based applications, we can develop targeted conservation strategies that harness the potential of these stress antagonists to mitigate the impacts of climate change on coral reefs.

## Supplementary Information

Below is the link to the electronic supplementary material.

Supplementary Material 1

Supplementary Material 2

## Data Availability

All raw data used for the results and figures in this manuscript is available in the Supplementary Table S2 including an explanation of the different parameters. Additionally, the calculated mean (percentage change) values reported in the results section are summarized in Supplementary Table S3.
