# Supplementary material for: Evaluating stress antagonists for enhanced coral recovery after natural heat exposure
Source: Sci Rep. 2025 Oct 9;15:35235. doi: 10.1038/s41598-025-22120-0 (PMC12511617; doi:10.1038/s41598-025-22120-0)
Supplement: Supplementary file 2 — Supplementary Material 2 [file 41598_2025_22120_MOESM2_ESM.docx]

Supplementary material to

**Evaluating Stress Antagonists for Enhanced Coral Recovery after natural Heat Exposure**

*Selma D. Mezger^1*^, Yusuf C. El-Khaled^2^, Susana Carvalho^2^, Raquel S. Peixoto^2^, Christian Wild^1^*

*^1^University of Bremen, Faculty of Biology and Chemistry, Marine Ecology Group, Leobener Str. 6, 28359 Bremen, Germany*

*^2^Red Sea Research Center (RSRC), Biological and Environmental Sciences and Engineering Division (BESE), King Abdullah University of Science and Technology (KAUST), Thuwal 23955, Saudi Arabia*

** corresponding authors: mezger@uni-bremen.de; susana.carvalho@kaust.edu.sa*

Information about Supplementary Tables File:

**Supplementary Table S1. Overview of antagonist treatments and heat stress on coral physiology found in the literature.** This table summarizes our four antagonist candidates (hydrogen, phosphate, ammonium, and probiotics) and their effects on coral physiology with and without additional heat stress, based on previous studies.

**Supplementary Table S2. Raw data for all physiological parameters measured in the experiment.** This table contains all raw measurements used for statistical analyses and figures in the manuscript. The dataset includes values for Survival, Coloration, Fv/Fm, chlorophyll *a* and c₂, protein content, oxygen fluxes, and the resulting P:R ratio. A description of all parameter names and units is provided in the "Explanation Table S2" .

**Supplementary Table S3. Calculated mean percentage changes for physiological parameters.** This table summarizes the mean percentage change for each parameter by species and treatment, as reported in the results section. These summary values correspond to the trends described in the manuscript and illustrated in the figures.


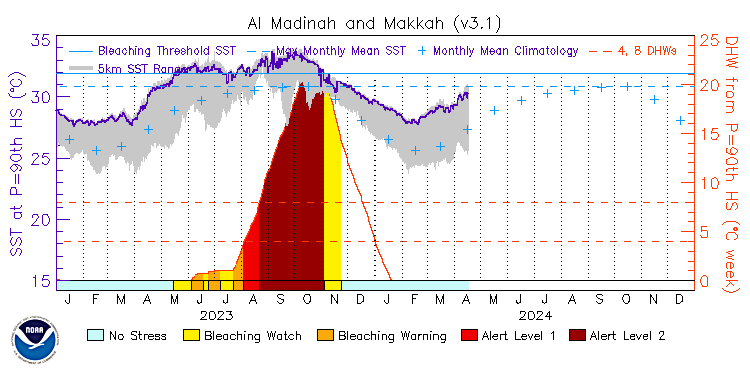


**Supplementary Figure S1.** **NOAA Coal Reef Watch Regional Virtual Station Time Series Graph for the region Al Madinah and Makkah in the year 2023 and 2024** (1)**.** The daily range of all SST values is shown in gray and maximum monthly mean as light blue dashed line. The 5km DHW is plotted as a red solid trace with the DHW scale (in °C-weeks) on the axis on the right. The colors that fill below the DHW trace correspond to the original Bleaching Alert Area heat stress levels. The 4 and 8 °C-weeks DHW thresholds are indicated with red dashed lines.


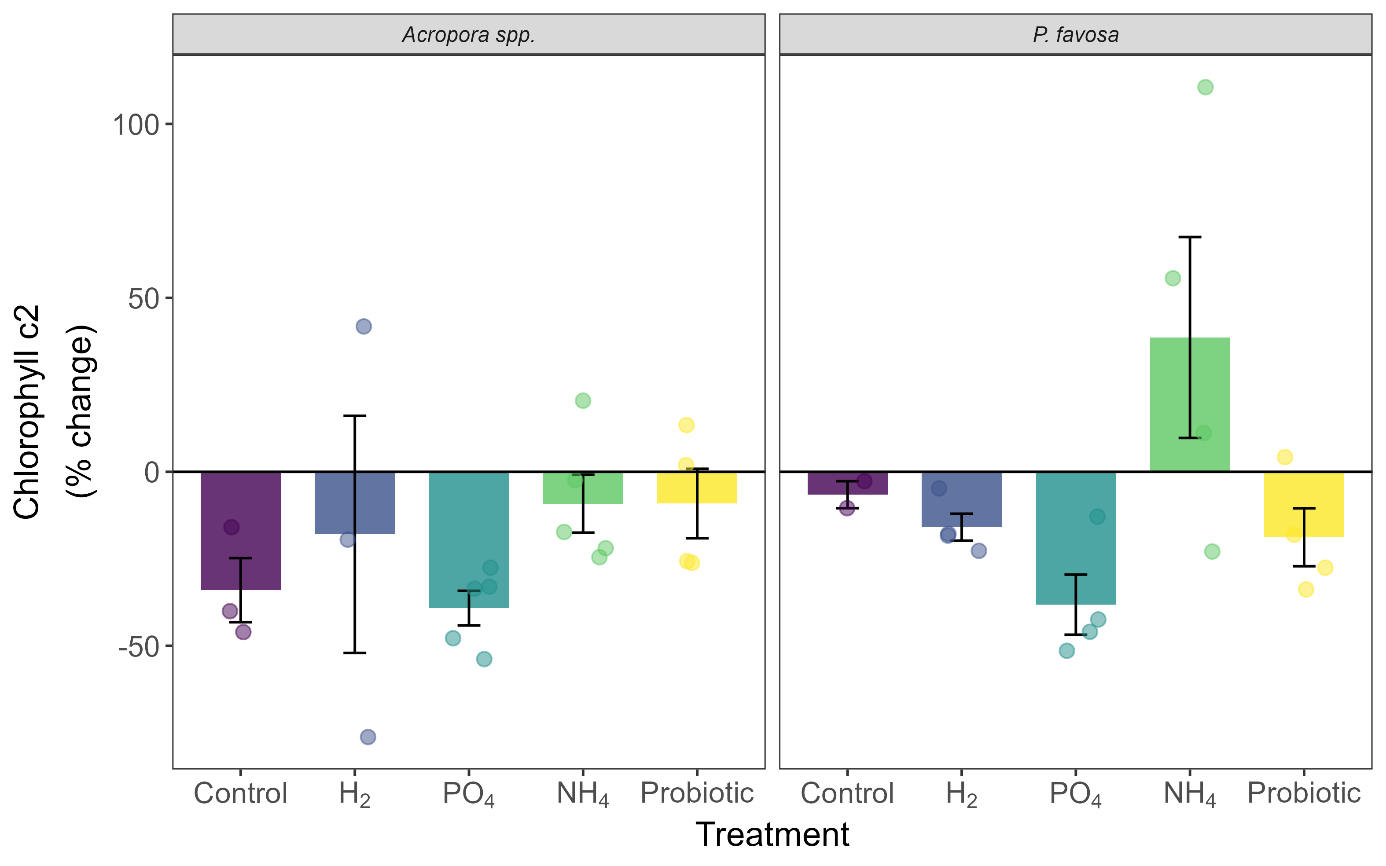


**Supplementary Figure S2.** **Percent change in Chlorophyll c2 concentration of *Acropora* spp. and *P. favosa* over the 48h experimental period.** Barplots show the mean % change with error bars representing the standard error. Each separate measurement is indicated by a dot in the respective treatment color.


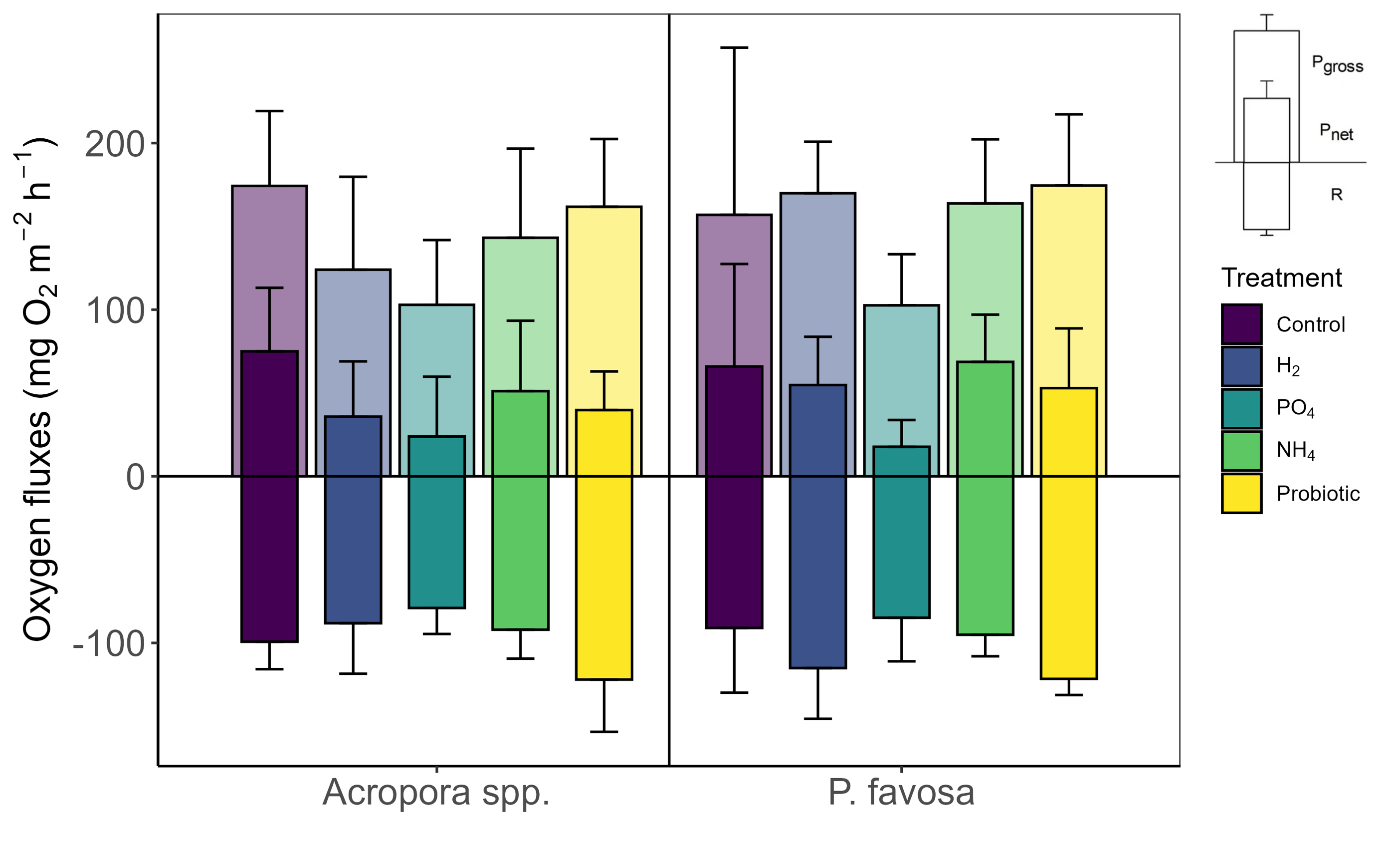


**Supplementary Figure S3.** **Gross- (P_gross_), net photosynthesis (P_net_), and respiration (R) of *Acropora* spp. and *P. favosa* after the 48h experimental period.** Error bars represent standard deviations.


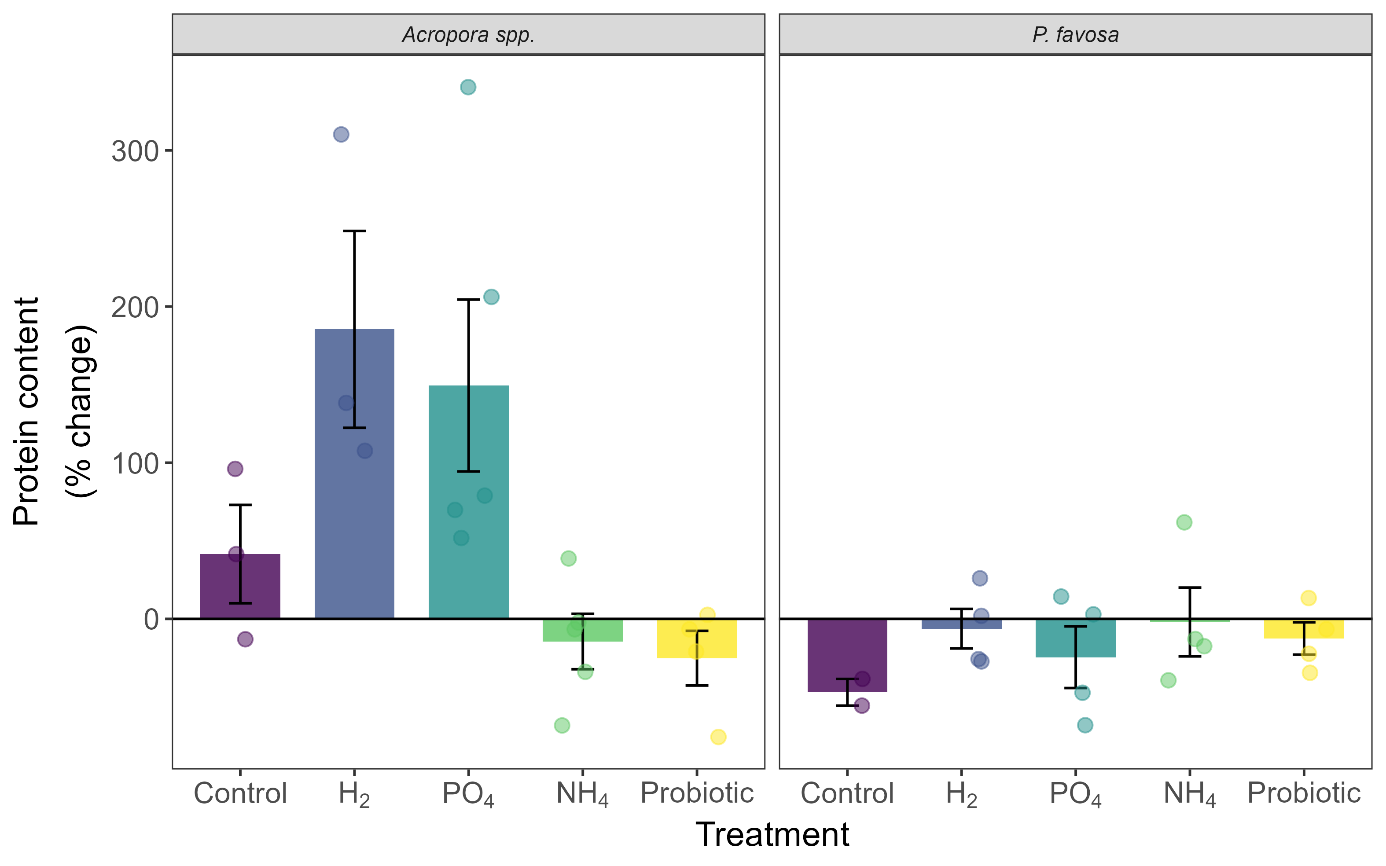


**Supplementary Figure S4.** **Percent change in protein content of *Acropora* spp. and *P. favosa* over the 48h experimental period.** Barplots show the mean % change with error bars representing the standard error. Each separate measurement is indicated by a dot in the respective treatment color.

**References**

1. NOAA Coral Reef Watch. Middle East 5 km Regional Virtual Station Time Series Graphs (Version 3.1, released July 31, 2019, experimental product). Graph accessed 2024-04-19 at https://coralreefwatch.noaa.gov/product/vs/timeseries/middle_east.php. 2024.
